# Combined treatment of Ketogenic diet and propagermanium reduces neuroinflammation in Tay-Sachs disease mouse model

**DOI:** 10.1007/s11011-025-01553-6

**Published:** 2025-02-28

**Authors:** Orhan Kerim Inci, Volkan Seyrantepe

**Affiliations:** 1 Izmir Institute of Technology, Department of Molecular Biology and Genetics, Gulbahce Mah, Izmir 35430 Urla, Turkey; 2 Izmir Institute of Technology, IYTEDEHAM, Gulbahce Mah, İzmir 35430 Urla, Turkey

**Keywords:** Standard Diet, Ketogenic Diet, Mouse model, Tay-Sachs disease, Neuroinflammation, Propagermanium

## Abstract

Tay-Sachs disease is a rare lysosomal storage disorder caused by β-Hexosaminidase A enzyme deficiency causing abnormal GM2 ganglioside accumulation in the central nervous system. GM2 accumulation triggers chronic neuroinflammation due to neurodegeneration-based astrogliosis and macrophage activity with the increased expression level of *Ccl2* in the cortex of a recently generated Tay-Sachs disease mouse model *Hexa-/-Neu3-/-*. Propagermanium blocks the neuroinflammatory response induced by Ccl2, which is highly expressed in astrocytes and microglia. The ketogenic diet has broad potential usage in neurological disorders, but the knowledge of the impact on Tay-Sach disease is limited. This study aimed to display the effect of combining the ketogenic diet and propagermanium treatment on chronic neuroinflammation in the Tay-Sachs disease mouse model. *Hexa-/-Neu3-/-* mice were placed into the following groups: (i) standard diet, (ii) ketogenic diet, (iii) standard diet with propagermanium, and (iv) ketogenic diet with propagermanium. RT-PCR and immunohistochemistry analyzed neuroinflammation markers. Behavioral analyses were also applied to assess phenotypic improvement. Notably, the expression levels of neuroinflammation-related genes were reduced in the cortex of 140-day-old *Hexa-/-Neu3-/-* mice compared to β-Hexosaminidase A deficient mice (*Hexa-/-*) after combined treatment. Immunohistochemical analysis displayed correlated results with the RT-PCR. Our data suggest the potential to implement combined treatment to reduce chronic inflammation in Tay-Sachs and other lysosomal storage diseases.

**Supplementary Information:**

The online version contains supplementary material available at 10.1007/s11011-025-01553-6.

## Introduction

Tay-Sachs disease (TSD) is a rare lysosomal storage disorder (LSD) that results from loss-of-function mutations in gene *HEXA*, which code for the α-subunits of the heterodimeric enzyme β-Hexosaminidase A (Hexa) (Leal et al. 2020). Hexa is a lysosomal glycosidase that degrades GM2 ganglioside (GM2) to GM3 ganglioside in brain and peripheral nervous system neurons with the assistance of an additional lysosomal protein called the GM2 activator (Toro et al. 2021). Infants with TSD are healthy when born. However, progressive accumulation of the GM2 ganglioside in neurons causes loss of neuromotor function, tremors, dystonia, and death in early childhood (Sandhoff and Harzer 2013). It has been reported that the TSD mouse model (*Hexa-/-*) has no phenotype for up to 1 year (Sango et al. 1995). On the other hand, we previously indicated the neuraminidase 3 (Neu3) bypass mechanism in a novel TSD mouse model *(Hexa-/-Neu3-/-)* by identifying Neu3 as the sialidase responsible for converting GM2 to GA2 (Seyrantepe et al. 2018). *Hexa-/-Neu3-/-* mice were healthy at birth; however, they showed severe neurological phenotype due to progressive accumulation of GM2 ganglioside and died at five months of age. Ataxia and tremor were among the neurological abnormalities. Behavioral analysis, including passive-avoidance, rotarod, and water maize, revealed age-dependent neuromotor coordination impairment and memory deficit, which indicated progressive neurodegeneration and neuroinflammation in *Hexa-/-Neu3-/-* compared to *Hexa-/-* mice.

It has been reported that ganglioside accumulation in lysosomes triggers the secretion of pro-inflammatory cytokines/chemokines from microglia and astrocytes, leading to neuroinflammation such as Gaucher disease (Hong et al. 2006), MPS type I and III (Archer et al. 2014), Niemann Pick disease type C (Cougnoux et al. 2018), neuronal ceroid lipofuscinosis (Francelle and Mazzulli 2022) and multiple sulfatase disease (Di Malta et al. 2012). In particular, it has been demonstrated that the accumulation of gangliosides in mice models of GM1 and GM2 gangliosides induces activation of microglia (microgliosis), astrocyte (astrogliosis), and the release of pro-inflammatory cytokines and chemokines such as Ccl2, Ccl3, and Cxcl10 (Jeyakumar et al. 2003; Akyıldız Demir et al. 2020).

Secreted Ccl2, which plays an essential role in the CNS mainly through interactions between astrocytes and microglia (Xu et al. 2017). It has been reported that abnormal Ccl2/Ccr2 expression levels increase leukocytosis and neuroinflammation in Gaucher, Sandhoff, and Farber diseases (Vitner et al. 2012; Ogawa et al. 2018; Yu et al. 2018). On the other hand, Ogawa et al. showed that anti-inflammatory drugs such as istradefylline mitigate *Ccl2* expression in the Sandhoff mice microglia and astrocytes, which display a reduction of pro-inflammatory cytokines and chemokines secretion (Ogawa et al. 2018). The anti-inflammatory activity of propagermanium (PG), an organic germanium compound, has been shown to inhibit the Ccl2/Ccr2 axis in chronic inflammation-induced animal models (Yokochi et al. 2001).

The ketogenic diet (KD), a combination of a low-carbohydrate and high-fat diet, burns fat instead of carbohydrates. Mimicking the fasting form, the KD ensures that fats, rather than carbohydrates, are the primary energy source. After KD administration, fatty acids are converted into ketone bodies, which are βHB, AcAc, and Acetone, by liver metabolism and entering the bloodstream, triggering nutritional ketosis and participating in various subsequent physiological or pathological reactions (Kossoff and Hartman 2012). While the KD was initially used to treat incurable epilepsy, it is now used to treat various neurological diseases such as Alzheimer’s disease and Parkinson’s disease (Stafstrom and Rho 2012; Jang et al. 2023). As there is currently neither a cure nor an effective disease-modifying therapy for many neurodegenerative disorders, it is crucial to develop effective strategies for treatments. Apart from neurological disorders, the KD plays a beneficial role in various LSDs such as Sandhoff disease and Niemann-Pick disease type C patients and mice models to reduce or prevent harmful effects of accumulating lysosome-associated components (Denny et al. 2010; Höller et al. 2021).

In this study, we aimed to elucidate the preventive efficacy of combined KD and PG treatment against the development of neuroinflammation in the TSD mouse model.

## Materials and methods

### KD Intervention in *Hexa-/-* and *Hexa-/-Neu3-/-* mice

**Fig. 1:**
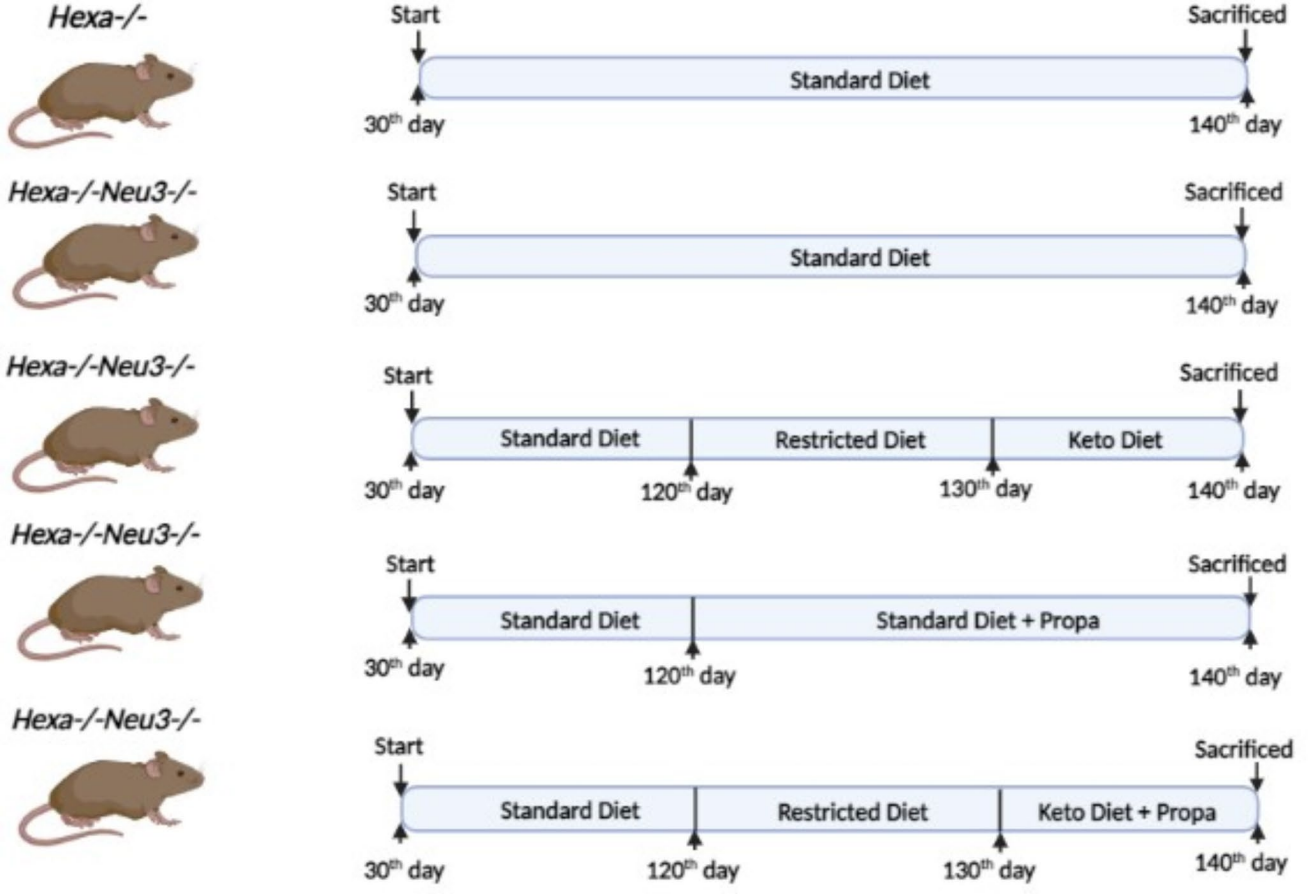
Illustration of the single and combined KD and PG treatment flow chart for the *Hexa-/-* and *Hexa-/-Neu3-/-* mouse model. *Hexa-/-* mice (*n* = 9) were used as controls and intervened with the standard diet. *Hexa-/-Neu3-/-* mice were fed until 140-day-old under the following groups: standard diet (SD) (*n* = 9), KD (10-day) (*n* = 9), SD with PG (8 mg/kg/daily for 20-day) (*n* = 9) and KD combined with PG (*n* = 9)

Breeding and maintenance of mice were supplied by the Turkish Council on Animal Care (TCAC)- an accredited animal facility of the Izmir Institute of Technology. The Animal Care and Use Committee of the Izmir Institute of Technology, Izmir, Turkey, granted the animal care approval. Animals were maintained at a constant temperature with an alternating 12-h light/dark cycle. Food and water were available ad libitum. All animal experiments were performed using the Turkish Institute of Animal Health guide for the care and use of laboratory animals. The Institutional Animal Care and Use Committee of the Izmir Institute of Technology approved the animal studies. Our study was designed in 140-day-old *Hexa-/-* and *Hexa-/-Neu3-/-* mice under the following groups: *Hexa-/-* with SD (*n* = 9), *Hexa-/-Neu3-/-* with SD (*n* = 9), *Hexa-/-Neu3-/-* with KD (10-day) (*n* = 9), *Hexa-/-Neu3-/-* with SD and PG (8 mg/kg/daily for 20-day) (*n* = 9) and *Hexa-/-Neu3-/-* with KD and PG (20-day) (*n* = 9) Fig. 1. Mice were separated on an individual basis to measure diet consumption and body weight accurately. Body weight was measured periodically once every three days until sacrifice. Ketone body level was calculated from the blood via tail vein phlebotomy using a Precision Xtra meter (Abbott Diabetes Care, Inc., cat no. #9881465) and ketone test strips (Abbott cat no. #7074565). The humane endpoint was defined by either weight loss of > 15% of body weight at the highest weight that the mouse reached or showed tremor. Animals were euthanized either by CO2 exposure followed by neck dislocation or by overdose of 0.25% Avertin (2,2,2-Tribromoethanol), followed by transcardial perfusion with PBS only or followed by 4% paraformaldehyde and tissue collection.

#### Gene expression analysis by real-time PCR

*Hexa-/-* and *Hexa-/-Neu3-/-* mice were sacrificed by cervical dislocation at 140 days old. Their brain cortices were separated and immediately frozen by liquid nitrogen. For the expression analysis of neuroinflammation-related genes (*Ccl2*, *Ccl3*, *Ccl5*, *Cxcl10*, and *Gfap*), RNA isolation from the cortex of *Hexa-/-* and *Hexa-/-Neu3-/-* mice (*n* = 3) was performed using Trizol Reagent according to the manufacturer’s instructions. Fifty ng/µl of cDNA was synthesized by the High-Capacity cDNA Reverse Transcription kit (BioRad, USA) according to the manufacturer’s instructions. The Real-Time PCR conditions were optimized using the LightCycler 96 machine (Roche, Switzerland) with 50 ng of cDNA in the 20 µl of reaction mix containing 0.4µM of primer pair and 1X LightCycler 480 SYBR Green I Master Mix (Roche, Switzerland). The conditions of PCR were denoted as in the following: 1 cycle of 10 min at 95 °C, 45 cycles of 20 s at 95 °C, 15 s at 57 °C, and 22 s at 72 °C. The values were read after each cycle. In the end, the amplification melting analysis was performed under the following conditions: 30 s at 95 °C, 10 s at 60 °C, then continuous reading while the temperature increased to 99 °C to detect the primer dimers, if any. The primers used to amplify the mRNA are listed in Table 1.

**Table 1:** List of primers used for quantitative RT-PCR analysis

Gene Names	Forward Primer	Reverse Primer
*Ccl2*	5’-ATGCAGTTAATGCCCCACTC-3’	5’-TTCCTTATTGGGGTCAGCAC-3’
*Ccl3*	5’-TCTGTACCATGACACTCTGC- 3’	5’- AATTGGCGTGGAATCTTCCG-3’
*Ccl5*	5’-AGTGCTCCAATCTTGCAGTC-3’	5’-AGCTCATCTCCAAATAGTTG-3’
*Cxcl10*	5’-ACCATGAACCCAAGTGCTGCCGT-3’	5’-AGGAGCCCTTTTAGACCTTTTTTG-3’
*Gfap*	5’-AGTAACATGCAAGAGACAGAG-3’	5’- TAGTCGTTAGCTTCGTGCTTG-3’
*B4Galnt1*	5’-GGGCGGTTGACCTCACTAAA-3’	5’-GGAGAACCGGACTGTGTCTG-3’
*B3Galt4*	5’-GGCAGTGCCCCTTCTGTATT-3’	5’-GTGCAGTCCTCTCCCCATTC-3’
*Gm3s*	5’-GCTGCCCGAACATGACTTTC-3’	5’-TGAAGTGCTTTGGCTGGAGT-3’
*Gd3s*	5’-AGGAGATTGTGCAAGGGGTG-3’	5’-TGGCGAATTATGCTGGGGTT-3’
*Hexb*	5’-AGTGCGAGTCCTTCCCTAGT-3’	5’-ATCCGGACATCGTTTGGTGT-3’
*Bcl-2*	5’-CGCAGAGATGTCCAGTCAGC-3’	5’-TATGCACCCAGAGTGATGCAG-3’
*Bcl-xL*	5’-TCAGCCACCATTGCTACCAG-3’	5’-GTCTGAGGCCACACACATCA-3’
*Bax*	5’-AGGATGCGTCCACCAAGAAG-3’	5’-CTTGGATCCAGACAAGCAGC-3’
*Gapdh*	5’-CCCCTTCATTGACCTCAACTAC-3’	5’-ATGCATTGCTGACAATCTTGAG-3’

### Immunohistochemical analysis

Coronal brain slices (10 μm thickness) from the mice at the indicated ages were treated with ice-cold acetone and then blocked using blocking buffer (4% BSA, 10% goat serum, 0.3% Triton X-100, and 0.3 M glycine in PBS), for one h at room temperature in a humidified chamber. anti-MOMA2 (1:50; ab33451; Abcam, USA) and anti-GFAP (1:200; ab7260; Abcam, USA) were diluted in a blocking buffer and applied overnight at 4 °C. The goat anti-rabbit Alexa Fluor 568 (Abcam, USA) conjugated secondary antibody was used to visualize primary antibodies. The slides were mounted with Fluoroshield mounting medium with DAPI (Abcam, USA), and images were obtained using a light microscope (Bx53, Olympus Corporation, Germany) equipped with a manually controlled specimen holder, a color camera, a fluorescent light source; and image analysis software (cellSens Entry, Olympus Corporation, Germany).

### Thin layer chromatography analysis

50 mg of brain tissue from each mouse was homogenized in 2 ml of dH_2_O using the ultraTurax homogenizer (IKA T10) for 30 s at 6000 rpm. The homogenized samples were sonicated (Bandelin-Sonoplus) for 3 min. The samples were dried with an N_2_ flow in the Reacti-Therm Heating module (Thermo) by 55 °C of water. The extraction was first performed with 3 ml of 100% acetone to remove the supernatant. Then, supernatants were collected via extraction with 1.5 ml of chloroform: methanol: water (10:10:1) solution and 2 ml of chloroform: methanol: water (30:60:8) solution. DEAE Sephadex A-25 was prepared freshly to separate the acidic gangliosides. The total ganglioside samples were loaded in the columns, washed with 4 ml of 100% methanol, and then acidic gangliosides were eluted with 5 ml of 500 mM potassium acetate in methanol. A desalting process of the acidic gangliosides was carried out by the Supelclean LC-18 column (Supelco). The collected acidic gangliosides were loaded in the LC-18 columns, and the flow through was discarded. The columns were washed with 10 ml of dH_2_O, and samples were eluted with 4 ml of methanol and 4 ml of chloroform: methanol (1:1) under low vacuum (< 15 Hg). The eluted samples were evaporated with an N_2_ flow. Samples were loaded onto silica plates (Merck) automatically (Linomat 5 Camag) and run in chloroform: methanol: 0.2% CaCl_2_ (30:65:8) solution. The plates were stained with 0.04 g orcinol (Sigma) dissolved in 10 ml of 25% sulfuric acid and incubated on a TLC plate heater (Camag) at 120 °C until all the bands became visible. The plates were then scanned with the HP scanner system, and the band intensities were analyzed using the ImageJ v1.54 program (Fiji).

### Behavioral analysis

The balance and coordination abilities were tested by rotarod test only in 140-day-old *Hexa-/-* and *Hexa-/-Neu3-/-* mice. Firstly, mice were trained on the rotarod unit at 4 rpm. After training, mice were tested on the accelerated rotarod unit from 4 to 40 rpm over 5 min. Three trials were carried out for each animal, and the mice’s duration times were recorded on the accelerated unit. Before testing each animal, the rotarod units were cleaned using 70% EtOH.

The open field test is a sensorimotor test conducted in a box with a 40 × 40 cm surface area and transparent walls. Anxiety behaviors of KD and PG groups of *Hexa-/-Neu3-/-* mice were studied compared with the same age groups of *Hexa-/-* mice. A digital camera was placed directly on top of the box. Mice were put into a corner of the box to roam in the area undisturbed for 5 min. Behavioral differences were analyzed using the Panlab SMART Video Tracking System v0.3 (Harvard Apparatus, USA).

## Results

### The KD slows down excessive weight loss in *Hexa-/-Neu3-/-* mice

**Fig. 2:**
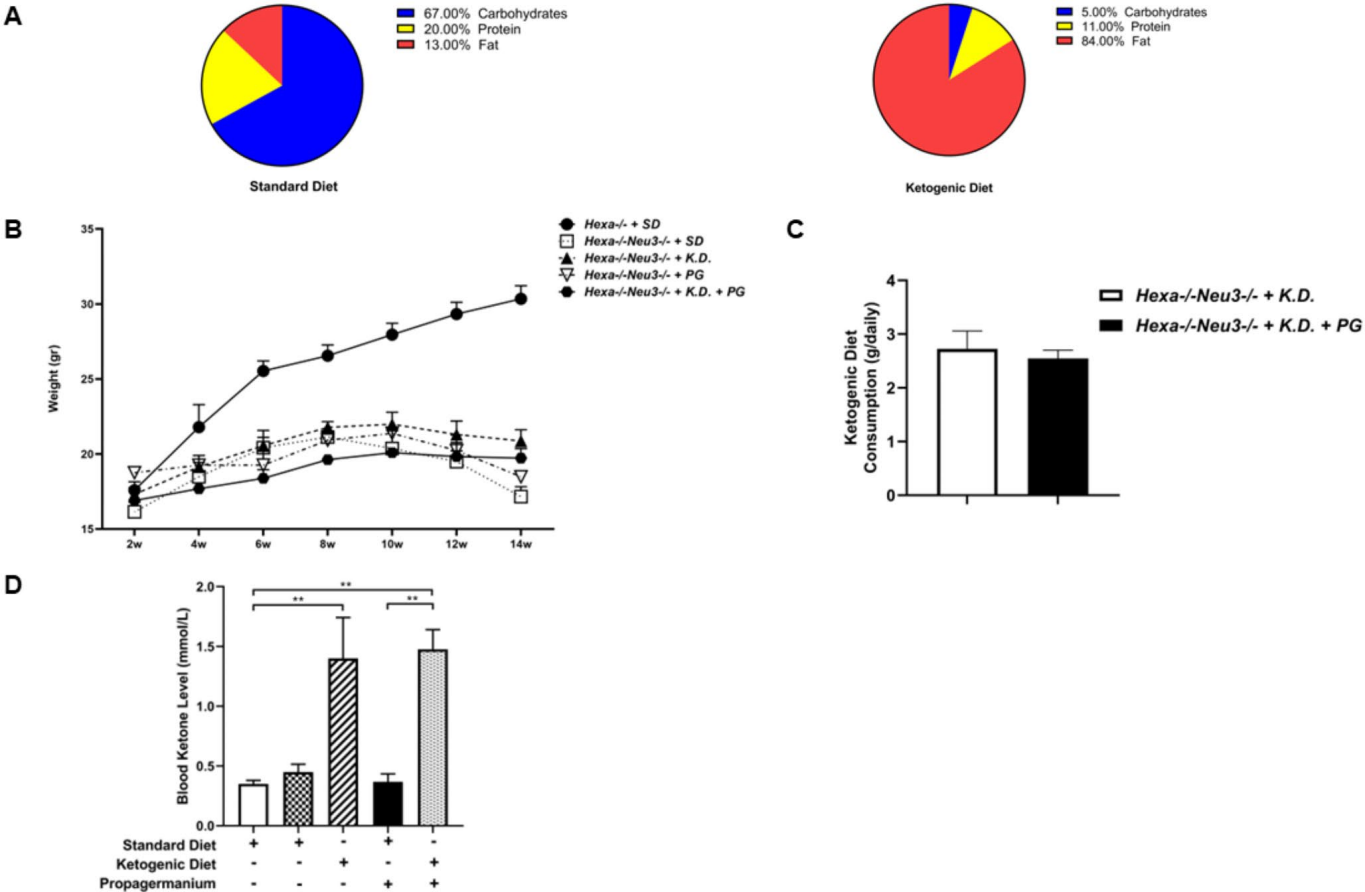
Composition of the intervented diet and their effects in the *Hexa-/-* and *Hexa-/-Neu3-/-* mouse model. Mice in each diet group intervened with the SD for 130 days and a KD for 10 days. The contribution of the data was shown here (*n* = 9 for the SD and KD, respectively). SD content comprises 67% carbohydrates, 20% protein, and 13% fat; KD content comprises 5% carbohydrates, 11% protein, and 84% fat (**A**). Body weight measurements of *Hexa-/-* (*n* = 9) and *Hexa-/-Neu3-/-* which was intervented SD (*n* = 9), ad-libitum KD (10-day) (*n* = 9), ad-libitum SD with PG (8 mg/kg/daily for 20-day) (*n* = 9) and KD combined with PG (*n* = 9) (**B**). Illustration of daily KD consumption between KD therapy group and combined KD with PG therapy group for *Hexa-/-Neu3-/-* mice(**C**). Measurements of blood ketone levels (mmol/L) for *Hexa-/-* and *Hexa-/-Neu3-/-* mice (**D**). The data are represented as the mean ± SEM. One-way ANOVA was used for statistical analysis (**p < 0.01)

Diet calorie composition is derived mainly from fat for the KD. However, calorie composition is derived mainly from carbohydrates for the standard diet (Fig. 2A). KD intervention was not able to slow down *Hexa-/-Neu3-/-* mice’s excessive weight loss until the human endpoint. Therefore, *Hexa-/-Neu3-/-* mice demonstrated a decreased lifespan with the disease progressing (Fig. 2B). KD and KD + PG mice groups consumed similar quantities of the KD (KD: 2,72 ± 0,67 g/day; KD + PG: 2,55 ± 0,26 g/day) for ten days (Fig. 2C). *Hexa-/-Neu3-/-* mice which are in KD (*n* = 9) and KD + PG (*n* = 9) treatment groups, displayed elevation of blood βHB (β-hydroxybutyrate) levels compared to SD-intervened *Hexa-/-Neu3-/-* mice from 0,45 ± 0,129 mM to 1,4 ± 0,683 mM and 0,45 ± 0,129 mM to 1,475 ± 0,33 mM respectively (Fig. 2D).

### Combined treatment significantly reduces expression levels of chemokines in the cortex

**Fig. 3:**
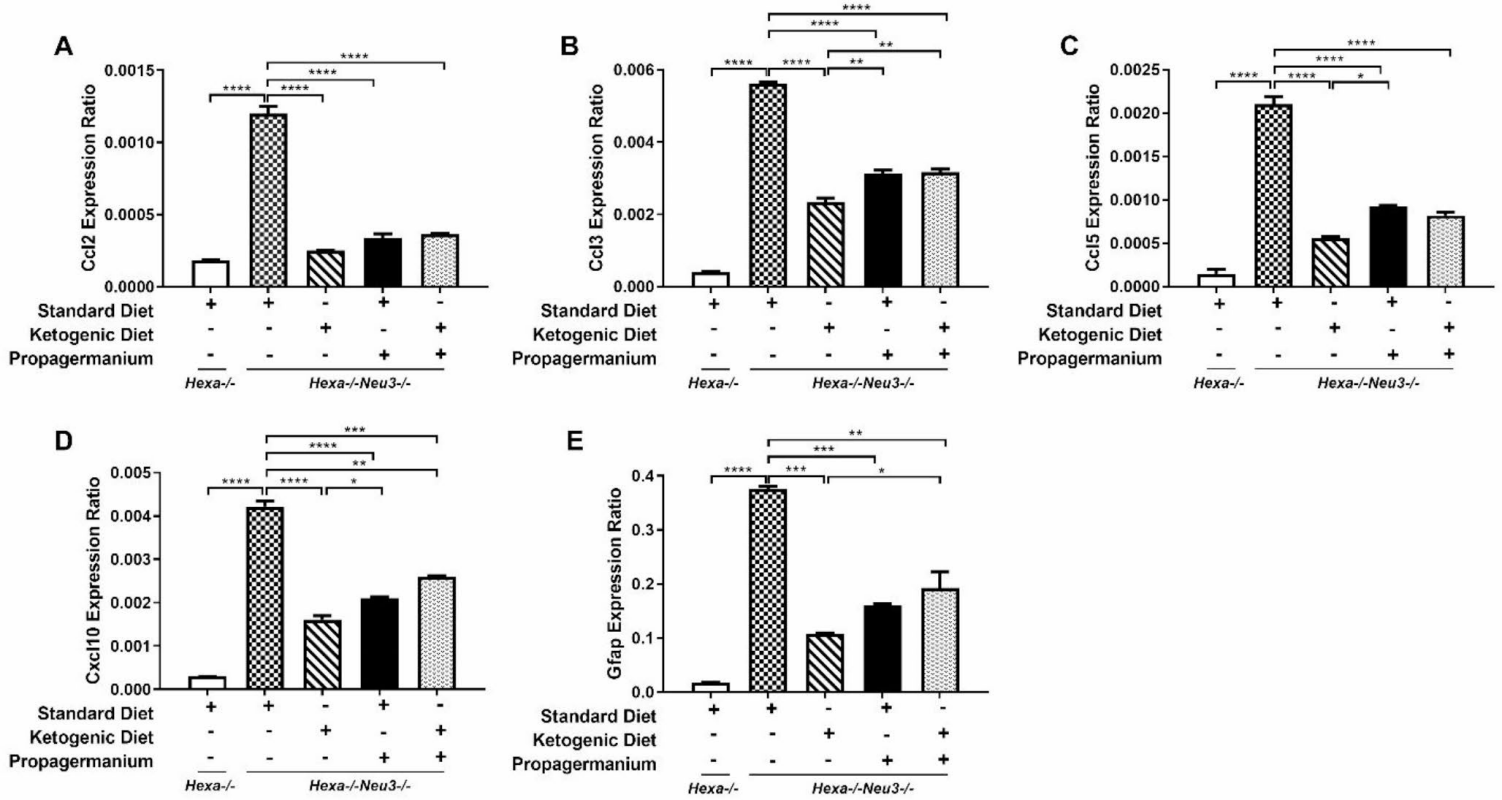
Relative expression levels of *Ccl2* (**A**), *Ccl3* (**B**), *Ccl5* (**C**), *Cxcl10* (**D**), and *Gfap* (glial fibrillary associated protein) (**E**) genes in the cortex for each diet group of 140-day-old *Hexa-/-* and *Hexa-/-Neu3-/-* mice. The data are represented as the mean ± SEM. One-way ANOVA was used for statistical analysis (**p < 0.01, *** *p* < 0.005 and ****p < 0.001) (*n* = 3)

**Fig. 4:**
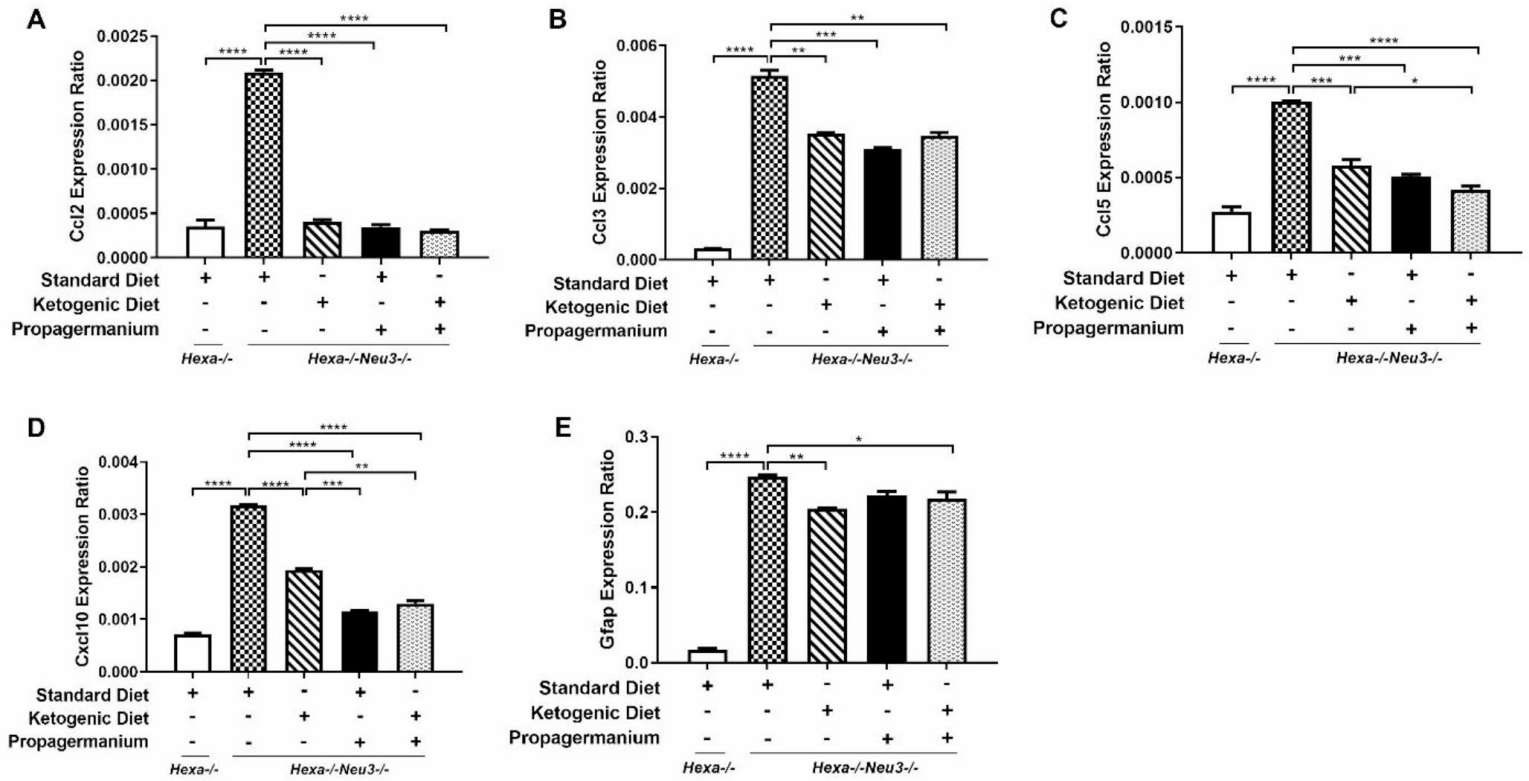
Relative expression levels of *Ccl2* (**A**), *Ccl3* (**B**), *Ccl5* (**C**), *Cxcl10* (**D**), and *Gfap* (glial fibrillary associated protein) (**E**) genes in the cerebellum for each diet group of 140-day-old *Hexa-/-* and *Hexa-/-Neu3-/-* mice. The data are represented as the mean ± SEM. One-way ANOVA was used for statistical analysis (**p < 0.01, *** *p* < 0.005 and ****p < 0.001) (*n* = 3)

Neuroinflammation triggers the secretion of pro-inflammatory cytokines/chemokines from macrophages in astrocyte and microglial cells. Our results indicated that 140-day-old *Hexa-/-Neu3-/-* mice displayed elevation of pro-inflammatory cytokine/chemokines gene expressions in the cortex. It is known that Ccl2 or Mcp-1(monocyte chemoattractant protein-1) is a cytokine involved in regulating immune cell infiltration and blood-brain barrier integrity (Cazareth et al. 2014; Yao and Tsirka 2014). At the same time, Ccl3 plays an influential role in the movement of lymphocytes, monocytes, and neutrophils with Ccl2 (Reichel et al. 2009). Ccl5, or RANTES, also assists the chemotaxis of T-cells and monocytes by interacting with various receptors (Murooka et al. 2008). In addition, Cxcl10 is another pro-inflammatory chemokine secreted by astrocytes that facilitates T-lymphocyte migration to tissue damage (Klein et al. 2005). We showed that all treatments significantly reduced gene expression levels of pro-inflammatory cytokines and chemokine (*Ccl2*, *Ccl3*, *Ccl5*, *Cxcl10*) and astrocyte markers (*Gfap*) in the cortex of *Hexa-/-Neu3-/-* mice (Fig. 3A-E). When each treatment was evaluated, except for the level of *Ccl2* expression, the most effective treatment in the cortical region was KD-only treatment. In parallel, a statistically significant decrease in the expression levels of neuroinflammation-related genes was also observed in the cerebellum of *Hexa-/-Neu3-/-* mice after KD and PG treatments, similar to the cortex (Fig. 4A-E). Furthermore, only the expression levels of *Ccl5* and *Cxcl10* genes were significantly reduced after combined treatment of KD and PG compared to KD alone (Fig. 4C-D). In addition to the neuroinflammatory role of chemokines, they also play essential roles in regulating neuronal death (Ramesh et al. 2013). For example, the Ccl2-knockout mouse model protects the neuron from TD (thiamine deficiency)-induced cell death due to macrophage/microglial activation (Yang et al. 2011). Furthermore, the expression level of *Ccl5* (*RANTES*) was increased in *Hexa-/-Neu3-/-* mice, similar to mouse models of the Niemann Pick type C (*Npc1-/-*), Gaucher (*Gba-/-)* and mucolipidosis type IV (*Mcoln1-/-)* (Cologna et al. 2014; Vitner et al. 2012; Cougnoux et al. 2019), and this increase was reduced after KD and PG treatments. Moreover, *Cxcl10* overexpression has been detected in neurodegenerative disorders such as Alzheimer’s disease and Parkinson’s disease (Zaheer et al. 2013; Kalkonde et al. 2007). KD treatment but no other treatments increased the expression level of anti-apoptotic gene expressions. Interestingly, not only Bax, one of the pro-apoptotic genes, expression level increased after KD treatment, but also *Bcl-2* and *Bcl-xL*, which are anti-apoptotic genes, expression levels increased (Fig. S1).

### The combined treatment alleviates astrogliosis in *Hexa-/-Neu*3*-/-* mice

Glia or neuroglia are the non-neuronal cells that mainly include astrocytes and microglia in the central nervous system. Microglial cells are resident immune cells with phagocytic activity to protect the central nervous system. Once activated, microglia move to damaged tissue and secrete pro-inflammatory cytokine/chemokine to scavenge dead cells by phagocytosis (Colonna and Butovsky 2017). In addition, active microglia trigger the macrophages/monocyte’s action and activation of astrocytes (Liddelow et al. 2017).

**Fig. 5:**
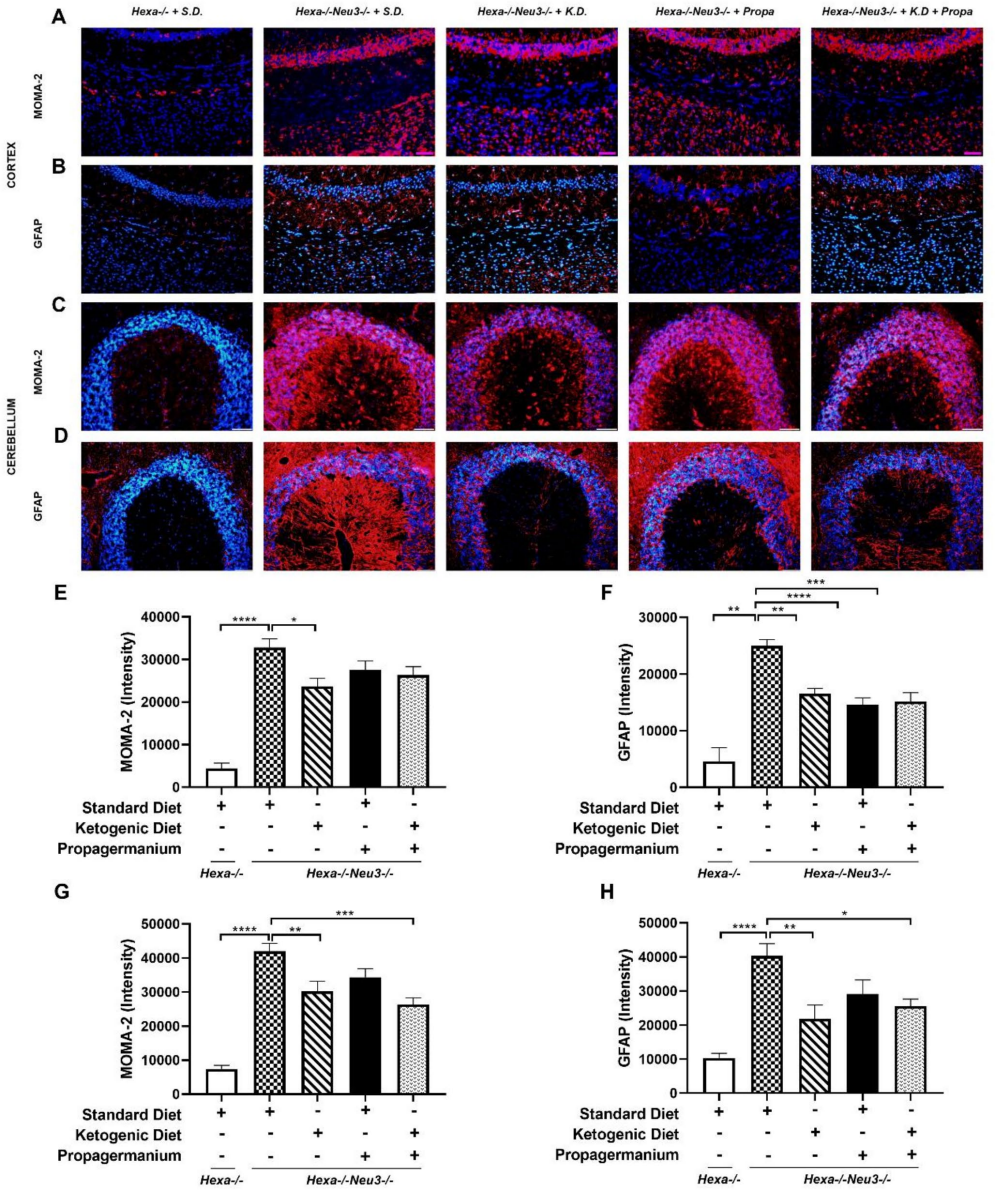
Immunofluorescent staining of anti-MOMA-2 (macrophage/monocyte) and anti-GFAP (astrocyte) in the cortex and cerebellum for each diet group of 140-day-old Hexa-/- and Hexa-/-Neu3-/- mice. Brain sections from the cortex and cerebellum, respectively, MOMA-2 (red) (**A**, **C**) and GFAP (red) (**B**, **D**) for each diet group of 140-day-old Hexa-/-Neu3-/- mice compared to Hexa-/- mice. Slides were mounted with DAPI (blue). Scale bar = 50 μm. Quantitative analysis of anti-MOMA-2 (**C**) and anti-GFAP (**D**) exhibits intensity that ImageJ detected. The data are represented as the mean ± SEM. One-way ANOVA was used for statistical analysis (**p < 0.01, ***p < 0.005 and ****p < 0.001) (*n* = 3)

It has been shown that the accumulation of undegraded macromolecules triggers a neuroinflammatory response in various LSDs (Vitner et al. 2010). Akyıldız-Demir et al. previously published that GM2 ganglioside accumulation induces neuroinflammation in the Tay-Sachs disease mouse brain cortex. KD and PG treatments did not alter the GM2 ganglioside level shown with thin-layer chromatography in the cortex (Fig S2); however, the gene expression level of enzymes involved in ganglioside biosynthesis was changed (Fig S3). To visualize the effects of a combined KD and anti-inflammatory drug treatment against neuroinflammation, 140-day-old *Hexa-/-* and *Hexa-/-Neu3-/-* mice brain sections were stained with anti-MOMA-2 and anti-GFAP which are specific for macrophage/monocyte and astrocyte respectively. MOMA-2-positive cells significantly increased in 140-day-old *Hexa-/-Neu3-/-* mice brain cortex compared to age-matched *Hexa-/-* mice (Fig. 5A-E). MOMA-2-positive cells were significantly reduced solely after KD treatment but not after anti-inflammatory drugs and combining KD and anti-inflammatory drug treatments. As in the cortex, a progressive increase in MOMA-2 positive cells is observed in the Hexa-/-Neu3-/- mice cerebellum. After KD and combined KD and PG treatments, not PG treatment, a significant decrease was observed in the cerebellum of *Hexa-/-Neu3-/-* mice (Fig. 5C-G). In contrast to MOMA-2 staining results, the GFAP-positive cells were approximately halved after the KD, anti-inflammatory, and a combination of the KD and anti-inflammatory drug treatments (Fig. 5B-F). In this study, KD treatment improved both astrogliosis and macrophage activity. However, a combined KD and PG did not significantly affect macrophage/monocyte activity in the 140-day-old Tay-Sachs mouse brain cortex. While an increase in astrocyte activation was also observed in the cerebellum of *Hexa-/-Neu3-/-* mice, parallel to that in the cortex, such an increase was rescued by KD and combined KD and PG treatments. However, no significant alteration was observed in PG treatment (Fig. 5D-H).

### Treatment with the KD and PG provides no improvement in anxiety-related behavior and motor activities

**Fig. 6:**
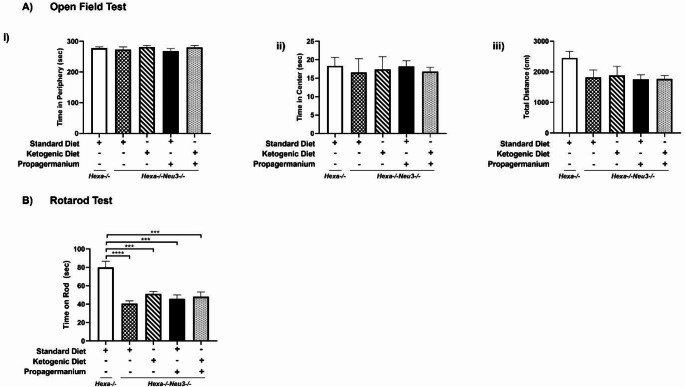
Anxiety behavior and locomotor activity of open field (**A**) and neuromotor activity of rotarod (**B**) were tested for each diet group of 140-day-old *Hexa-/-* and *Hexa-/-Neu3-/-* mice. Time spent in the periphery (i), the center (ii), and total distance (iii) in the open field area were analyzed. *Hexa-/-* (*n* = 9) and *Hexa-/-Neu3-/-* which was intervented standard diet (SD) (*n* = 9), ad-libitum KD (10-day) (*n* = 9), ad-libitum SD with PG (8 mg/kg/daily for 20-day) (*n* = 9) and KD combined with PG (*n* = 9). The data are represented as the mean ± SEM. One-way ANOVA was used for statistical analysis (***p < 0.005 and ****p < 0.001)

The open-field test was used to measure levels of anxiety-related behavior via mice’s body activity and locomotion. Combined KD and PG did not alter time spent in the periphery and center of the platform and total distance traveled for 140-day-old *Hexa-/-Neu3-/-* mice compared to age-matched *Hexa-/-* (Fig. 6A).

We previously showed that 140-day-old *Hexa-/-Neu3-/-* mice displayed a progressive reduction of neuromotor activity in the rotarod test. Since those mice could not stay on the accelerated rod for extended periods compared to age-matched *Hexa-/-* mice, our results indicated that each treatment did not improve neuromotor coordination and balance in *Hexa-/-Neu3-/-* mice (Fig. 6B).

## Discussions

Studies targeting neuroinflammatory processes involved in LSD’s mice models have been accelerated tremendously in recent years (Horii et al. 2022; Pan et al. 2024; Fyke et al. 2024). Previously, we reported that the *Hexa-/-Neu3-/-* mouse model displayed induced neuroinflammation due to the accumulation of the GM2 ganglioside in lysosomes, neural loss, and neurodegeneration (Akyıldız-Demir et al. 2020). Neuroinflammation is a common sign of LSDs because undegraded substrates trigger the secretion of the pro-inflammatory cytokine and chemokines and activate the macrophage, astrocyte, and microglia (Bosch and Kielian 2015; Pandey 2023). A limited number of studies have evaluated the effects of the combined KD and anti-inflammatory drug treatment on neuroinflammation, especially in LSDs (Denny et al. 2010).

The current study demonstrated that additive interaction between the KD and PG reduces neuroinflammation in the *Hexa-/-Neu3-/-* mice cortex. Our group previously published that GM2 accumulation causes the activation of macrophage and astrocytes in the *Hexa-/-Neu3-/-* mice cortex. Activation of the macrophage and astrocytes led to pro-inflammatory cytokine/chemokine secretions. We demonstrated that *Ccl2*, *Ccl3*, *Ccl5*, *Cxcl10*, and *Gfap* expression levels significantly increase in 4.5-month-old *Hexa-/-Neu3-/-* mice cortex compared to aged-matched *Hexa-/-* (Akyıldız-Demir et al. 2020). It has been shown that glucosylceramide accumulation induces inflammatory cytokine production from microglia in glucocerebrosidase-deficient mice (C57BL/6J-Gba^tm1Nsb^) (Hong et al. 2006). Moreover, in *NPC1-/-* mice, increased secretion of cytokines and chemokines such as TNF-α and IL-1β was observed as a result of microglial activation, whereas these secretions decreased after intraperitoneal administration of 2-Hydroxypropyl-β-cyclodextrin (HPβCD) for 6 weeks in 7-week-old animals (Cougnoux et al. 2018). Besides, astrocytes from the multiple sulfatase deficient mouse (*Sumf1-/-*) elicit aberrant accumulation of lysosomal storage that increases expression levels of *Mip1-α*, *Mip1-β*, and *TNF-α* in the brain tissue compared to control mice (Di Malta et al. 2012).

A CCR2 signaling blocker known as PG (bis(2-carboxyethyl germanium) sesquioxide) has been shown to have potent anti-inflammatory and immune-modulating capabilities (Lei et al. 2023). PG prevents ischemic brain damage by inhibiting neuroinflammation via Ccl2/Ccr2 (Tsukuda et al. 2011; He et al. 2019). In addition, Ogawa et al. showed that inhibition of the A2A receptor by istradefylline, which was administered daily for 7 days from 10 to 13 weeks of *Hexb−/−* mice, decreased the number of activated microglial cells and inflammatory cytokines/chemokines, especially Ccl2 (Ogawa et al. 2018). Ccl2/Ccr2 plays a prominent role, especially in microglial activation and neurotoxicity (Geng et al. 2022). In addition, the inactivation of Ccl2/Ccr2 as a mouse model or inhibiting the Ccl2/Ccr2 via molecular agents has been shown to prevent inflammation, cerebral blood barrier dysfunction, and neuronal apoptosis (Yang et al. 2011). The KD is a high-fat and low-glucose diet that triggers ketone body production from the liver due to low glucose levels. Biochemical analysis displayed that KD beneficial effects arise from the production of ketones such as β-hydroxybutyrate, acetoacetate, etc. (Jensen et al. 2020). The administration of KD and exogenous ketone supplements have recently been proposed as adjunctive therapy in neurodegenerative diseases (Camberos-Luna and Massieu 2020) and various lysosomal storage diseases (Denny et al. 2010; Nilsson et al. 2022; Stumpf et al. 2019). In addition, the KD treatment reveals that it can take advantage of neuroprotection and reduce inflammation in the body (Yang and Cheng 2010).

Our results indicate that the KD and combined KD and PG treatments reduce macrophage/astrocyte activation against GM2 accumulation and alleviate neuroinflammation-related gene expressions in the cortex and cerebellum, depending upon the anti-inflammatory characteristics of KD and PG. These findings suggest modifying pro-inflammatory chemokines or their receptors as prospective therapeutic targets to delay neuropathology in TSD.

## Electronic Supplementary Material

Below is the link to the electronic supplementary material.

Supplementary Material 1: Figure S1: Relative expression levels of anti-apoptotic *Bcl-2* (A) and *Bcl-xL* (B), pro-apoptotic *Bax* (C), genes in the cortex for each diet group of 140-day old *Hexa-/-* and *Hexa-/-Neu3-/- *mice. The data are represented as the mean ± SEM. Two-way ANOVA was used for statistical analysis (**p < 0.01 ) (*n*=3) 

High Resolution Image (TIF 561 KB)

Supplementary Material 2: Figure S2: Thin layer chromatography showing orcinol stained ganglioside profile extracted from cortex for each diet group of 140-day old *Hexa-/-* and *Hexa-/-Neu3-/- *mice (A). The histogram shows GM2/GD1a (B) and GM2/GM1 (C) intensity ratio. Intensities were measured via the ImageJ program. The data are represented as the mean ± SEM. One-way ANOVA was used for statistical analysis (*p <0.05 and **p<0.01) (*n*=3).

High Resolution Image (TIF 7.65 MB)

Supplementary Material 3: Figure S3:Relative expression levels of ganglioside synthesis and catabolism genes in the cortex for each diet group of 140-day-old *Hexa-/-* and *Hexa-/-Neu3-/- *mice. The data are represented as the mean ± SEM. One-way ANOVA was used for statistical analysis (*p <0.05, **p <0.01, ***p <0.005 and ****p<0.001) (*n*=3).

High Resolution Image (TIF 27.6 MB)

## Data Availability

No datasets were generated or analysed during the current study.
