# Supplementary figures and images for: Combined treatment of Ketogenic diet and propagermanium reduces neuroinflammation in Tay-Sachs disease mouse model
Source: Metab Brain Dis. 2025 Feb 28;40(3):133. doi: 10.1007/s11011-025-01553-6 (PMC11870964; doi:10.1007/s11011-025-01553-6)

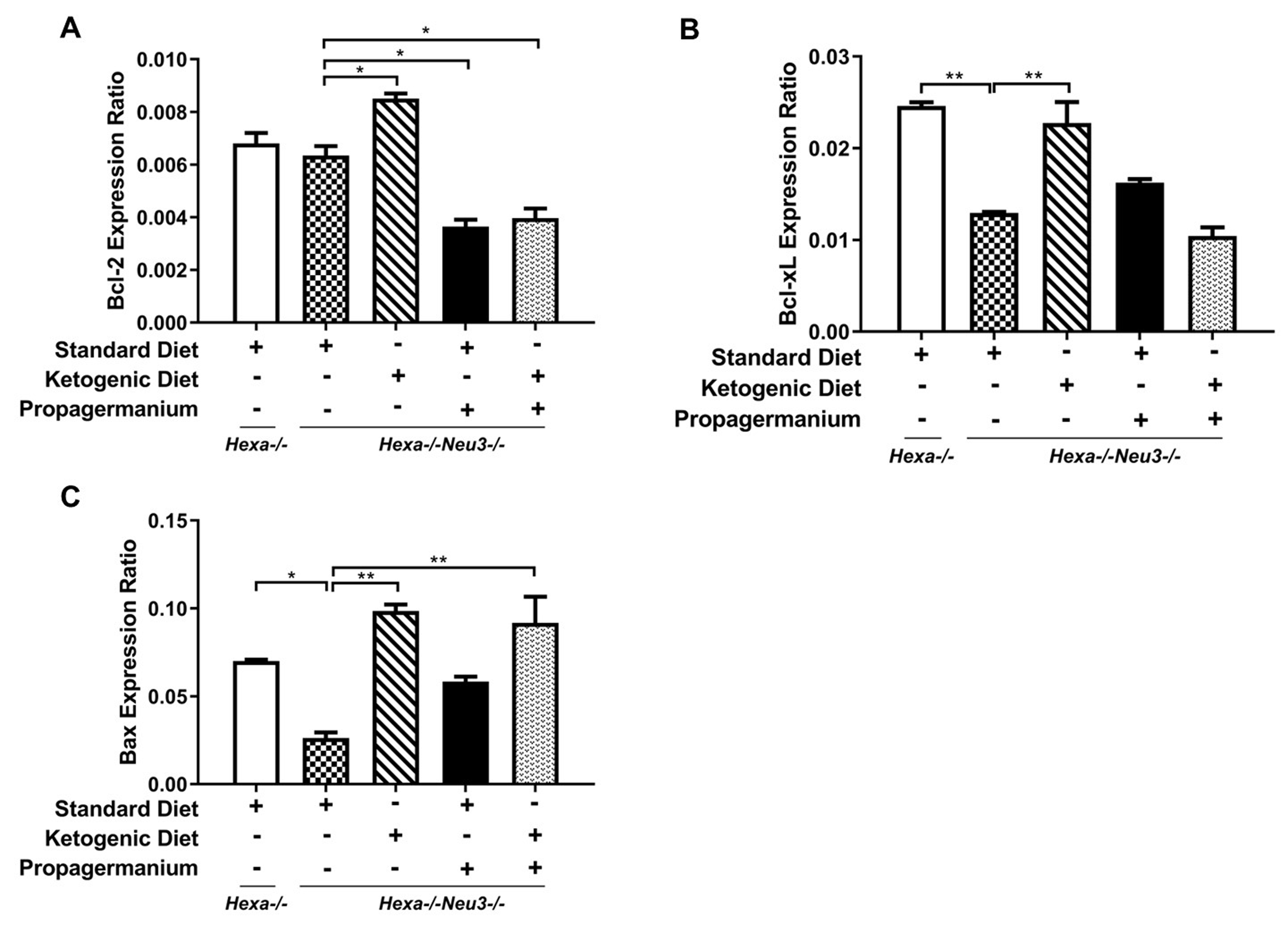

Supplement: Supplementary file 1 — Supplementary Material 1: Figure S1: Relative expression levels of anti-apoptotic Bcl-2 (A) and Bcl-xL (B), pro-apoptotic Bax (C), genes in the cortex for each diet group of 140-day old Hexa-/- and Hexa-/-Neu3-/- mice. The data are represented as the mean ± SEM. Two-way ANOVA was used for statistical analysis (**p < 0.01 ) (n=3) [file 11011_2025_1553_MOESM4_ESM.png]

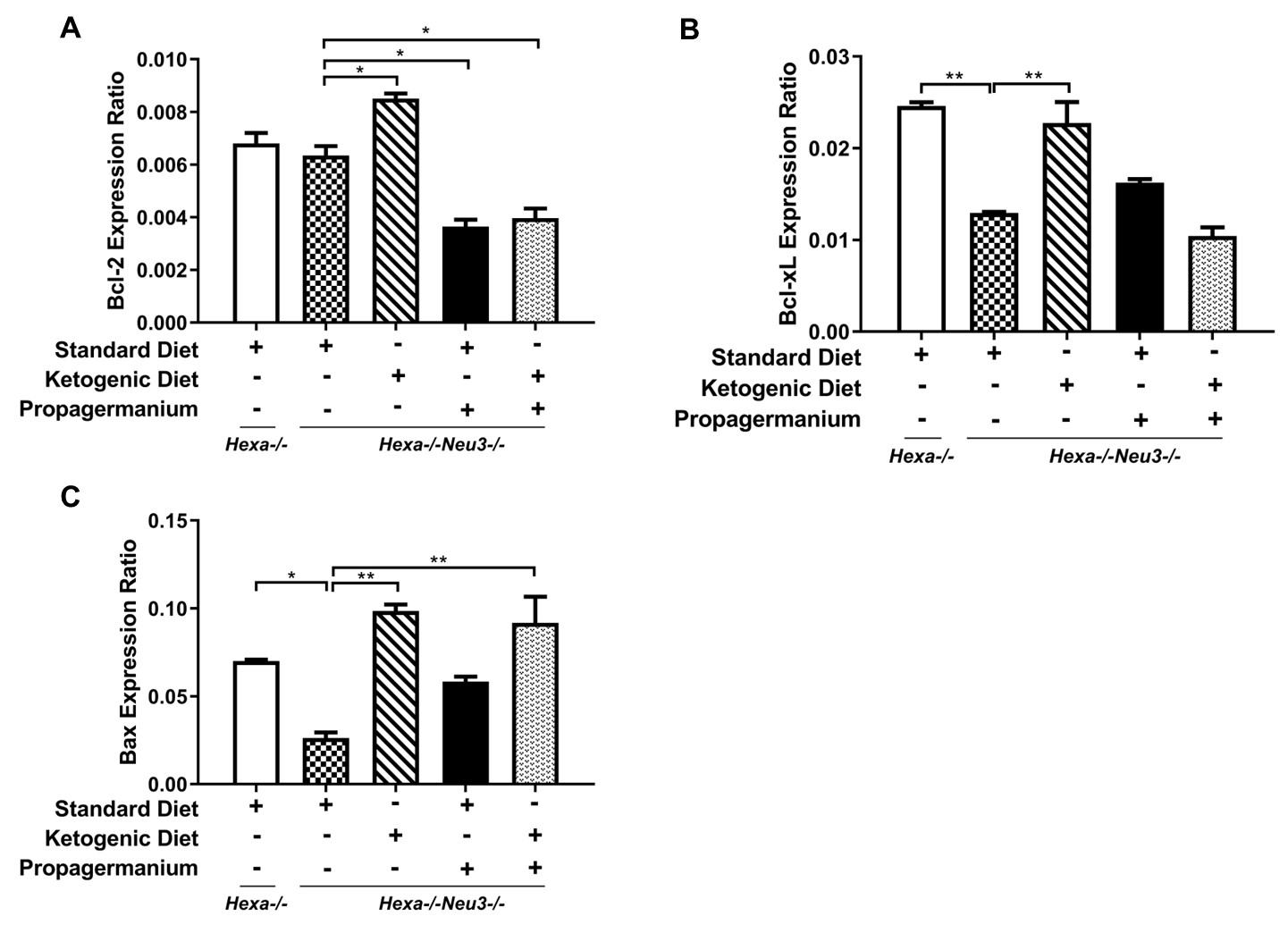

Supplement: Supplementary file 2 — High Resolution Image (TIF 561 KB) [file 11011_2025_1553_MOESM1_ESM.tif]

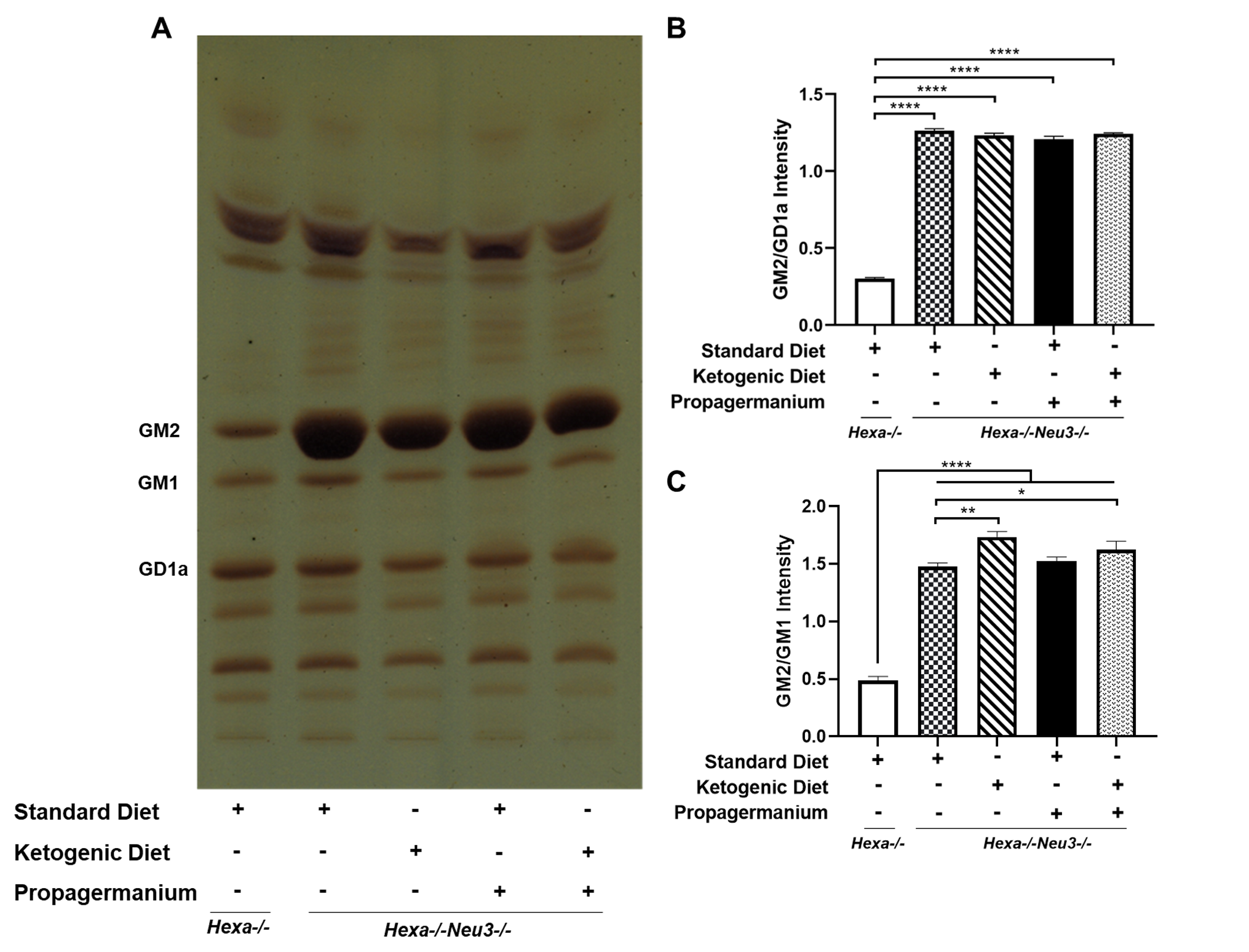

Supplement: Supplementary file 3 — Supplementary Material 2: Figure S2: Thin layer chromatography showing orcinol stained ganglioside profile extracted from cortex for each diet group of 140-day old Hexa-/- and Hexa-/-Neu3-/- mice (A). The histogram shows GM2/GD1a (B) and GM2/GM1 (C) intensity ratio. Intensities were measured via the ImageJ program. The data are represented as the mean ± SEM. One-way ANOVA was used for statistical analysis (*p <0.05 and **p<0.01) (n=3). [file 11011_2025_1553_MOESM5_ESM.png]

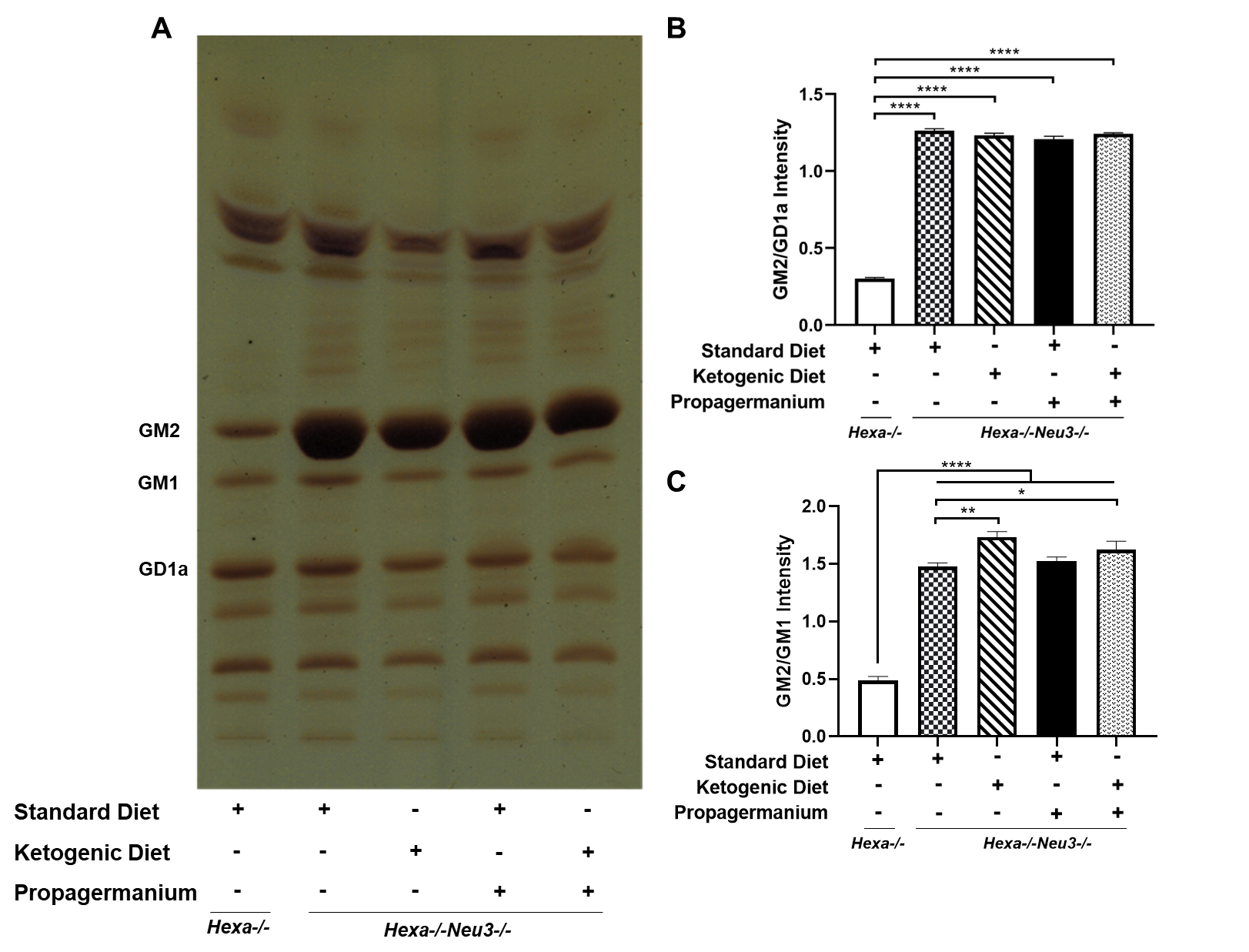

Supplement: Supplementary file 4 — High Resolution Image (TIF 7.65 MB) [file 11011_2025_1553_MOESM2_ESM.tiff]

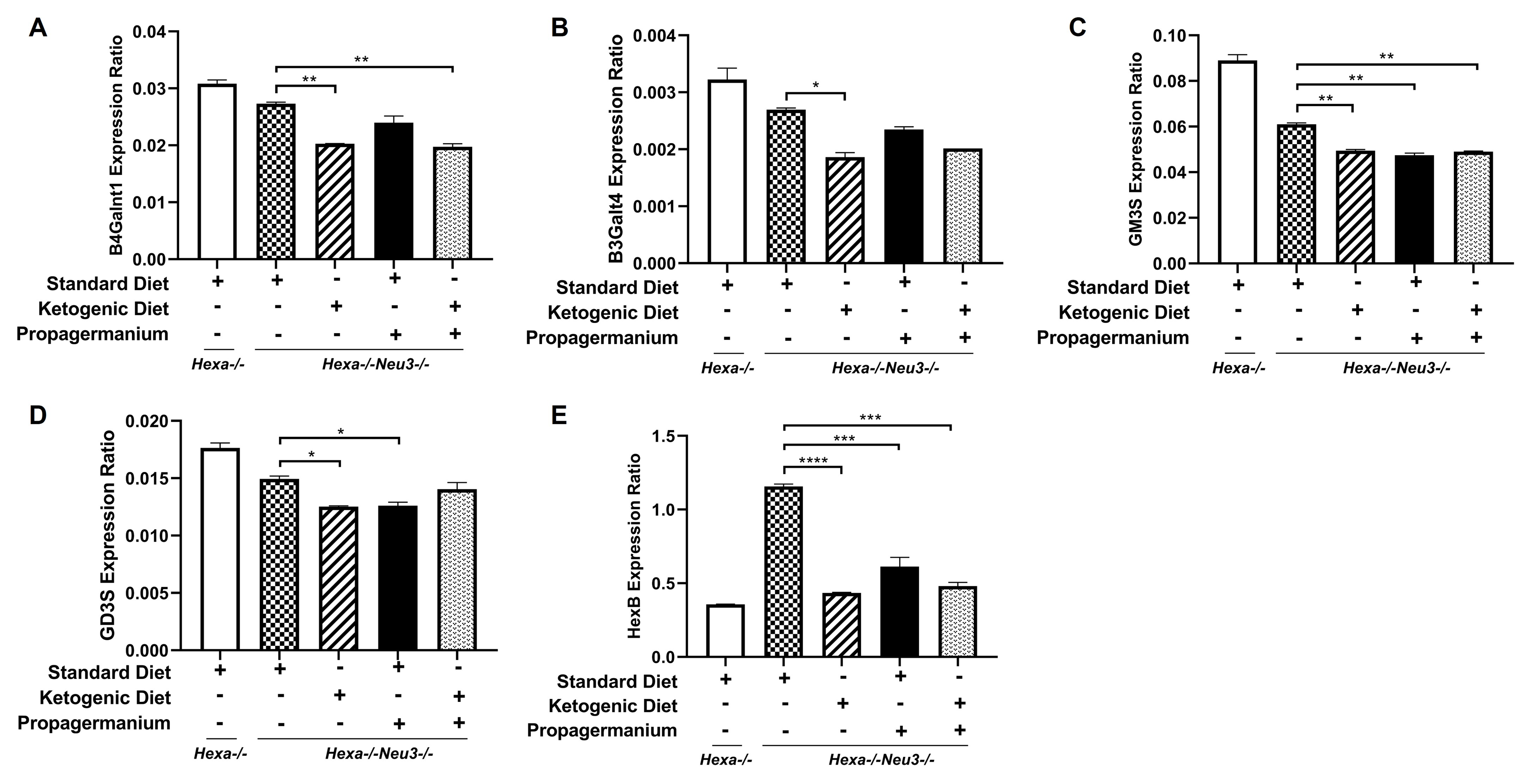

Supplement: Supplementary file 5 — Supplementary Material 3: Figure S3:Relative expression levels of ganglioside synthesis and catabolism genes in the cortex for each diet group of 140-day-old Hexa-/- and Hexa-/-Neu3-/- mice. The data are represented as the mean ± SEM. One-way ANOVA was used for statistical analysis (*p <0.05, **p <0.01, ***p <0.005 and ****p<0.001) (n=3). [file 11011_2025_1553_MOESM6_ESM.png]

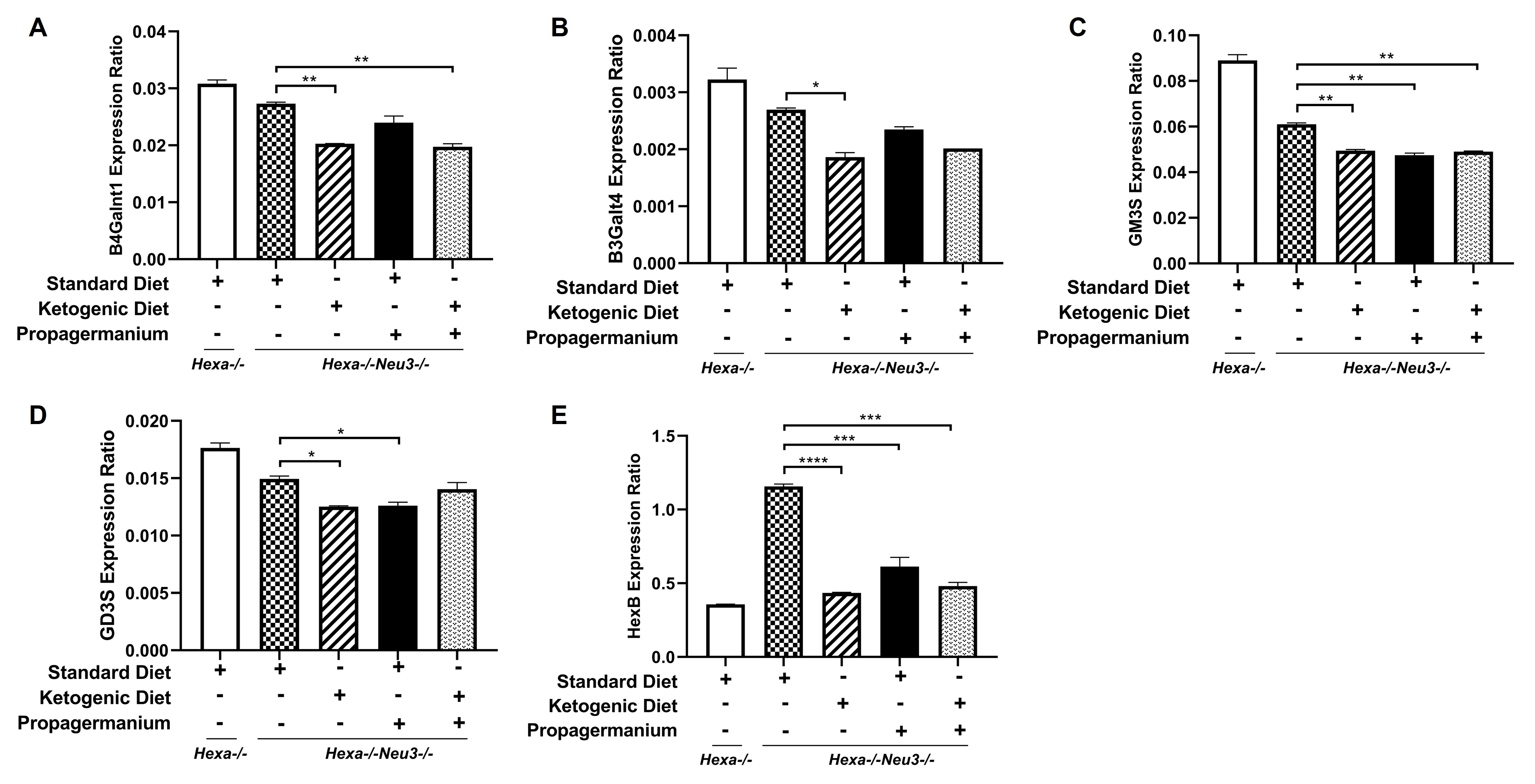

Supplement: Supplementary file 6 — High Resolution Image (TIF 27.6 MB) [file 11011_2025_1553_MOESM3_ESM.tiff]
